# Supplementary material for: CD109 Is a Critical Determinant of EGFR Expression and Signaling, and Tumorigenicity in Squamous Cell Carcinoma Cells
Source: Cancers (Basel). 2022 Jul 28;14(15):3672. doi: 10.3390/cancers14153672 (PMC9367592; doi:10.3390/cancers14153672)
Supplement: Supplementary file 1 [file cancers-14-03672-s001.zip › cancers-1753397-supplementary.pdf]

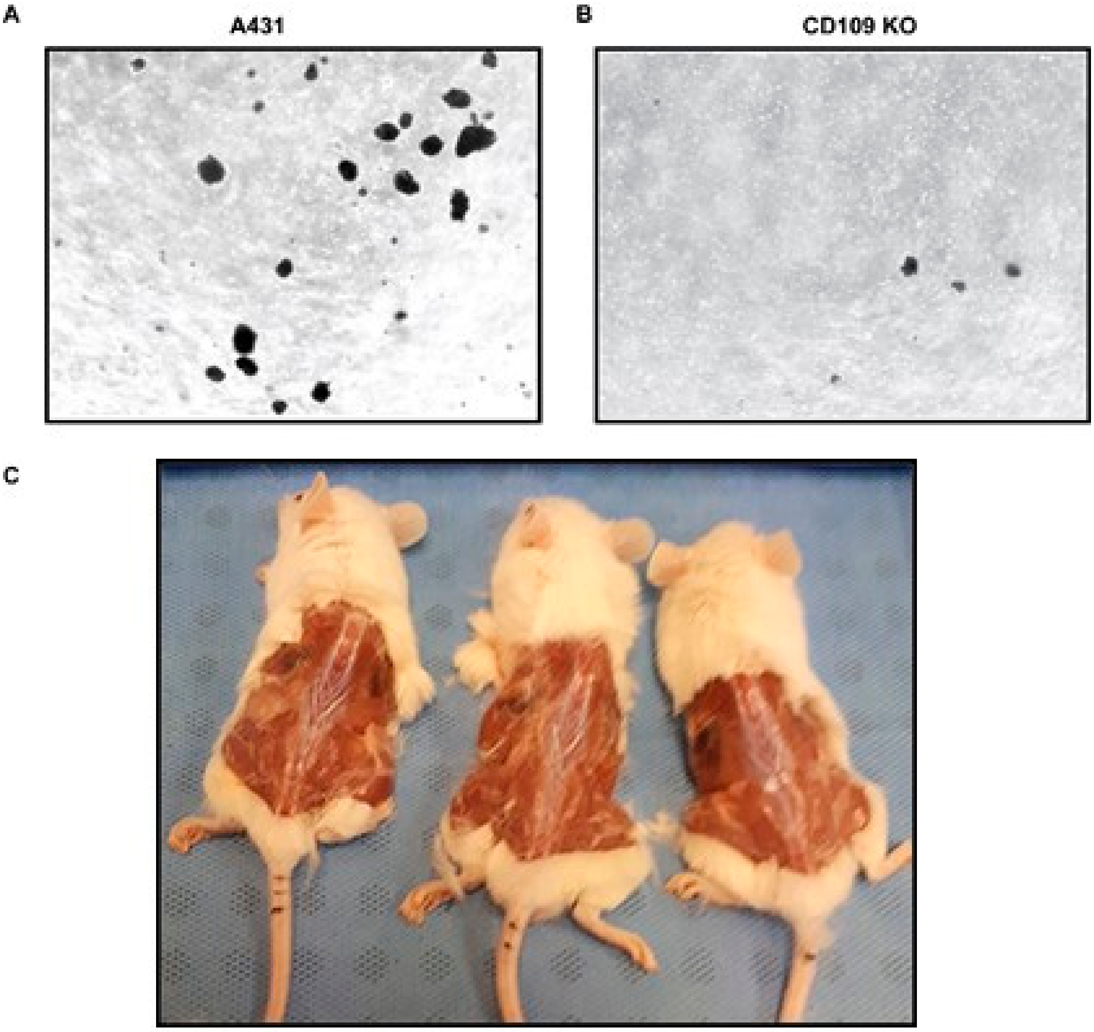

Figure S1: The loss of CD109 abrogates tumorigenicity of A431 cells (A) Representative phase contrast images for spheroids from A431 cells after the third passage. (B) Representative phase control images for spheroids from CD109 KO cells after the third passage, showing that CD109KO spheroids completely lost their ability to form spheroid after 3 passages. (C) Mice that received CD109-KO cells remained tumor free for another additional 6 months.

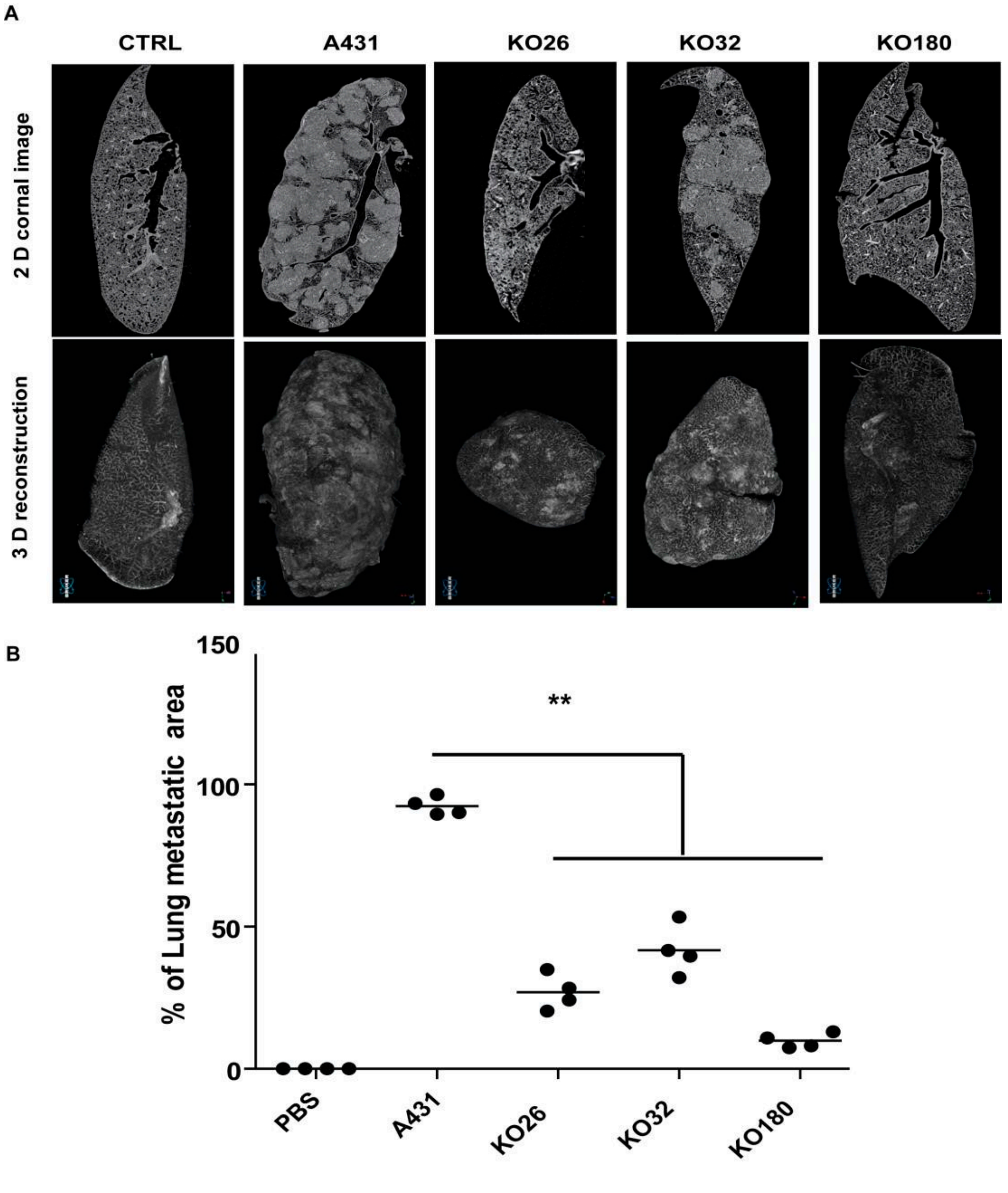

Figure S2: Micro-CT analysis of lung section (A) Representative images of Micro-CT analysis of lung sections in formalin-fixed lungs. (B) Quantification of lung metastasis from Micro -CT analysis in mice injected with PBS control, control A431 cells or one of the three CD109 KO cells. (n = 20). Lung metastases area in mice that received CD109-KO cells were significantly smaller compared to the metastatic burden produced by control A431 cells. All the results are expressed as the mean ± S.D. Significance is calculated using a One-Way ANOVA * $P < 0.05$. ** $P < 0.01$ and *** $P < 0.001$.

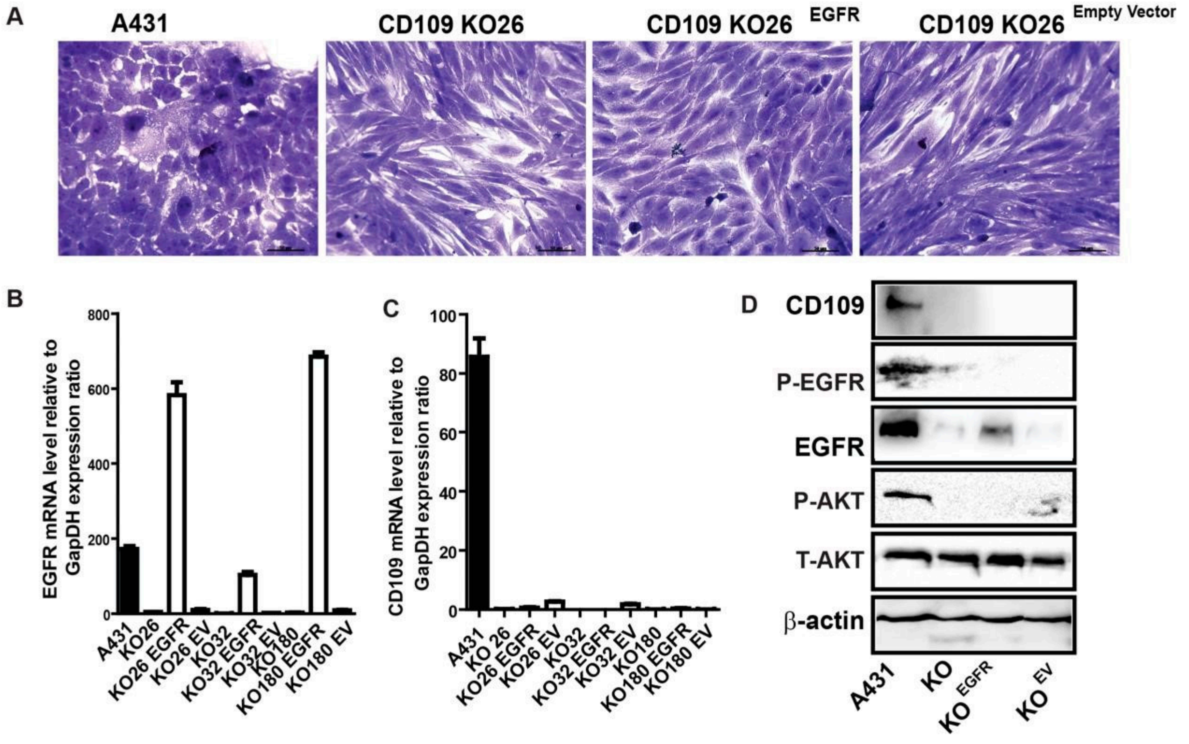

Figure S3: Overexpression of EGFR in CD109 KO cells (A) Representative images of toluidine blue staining of control A431 cells, or CD109KO A431 cells transfected with and without EGFR plasmid or empty vector, indicating that overexpression of EGFR alone does not affect the cell morphology of CD109KO cells**.** (B) Quantitative PCR analysis of EGFR expression in the cells as indicated. (C) Quantitative PCR analysis of CD109 expression in the cells as indicated. (D) Western blotting analysis of A431, CD109 KO, and CD109^{EGFR}cells with indicated antibodies. All the results are expressed as the mean ± S.D. of three independent experiments. Significance is calculated using a One-Way ANOVA * P < 0.05. ** P < 0.01 and *** P < 0.001. Scale bar=100 µm.

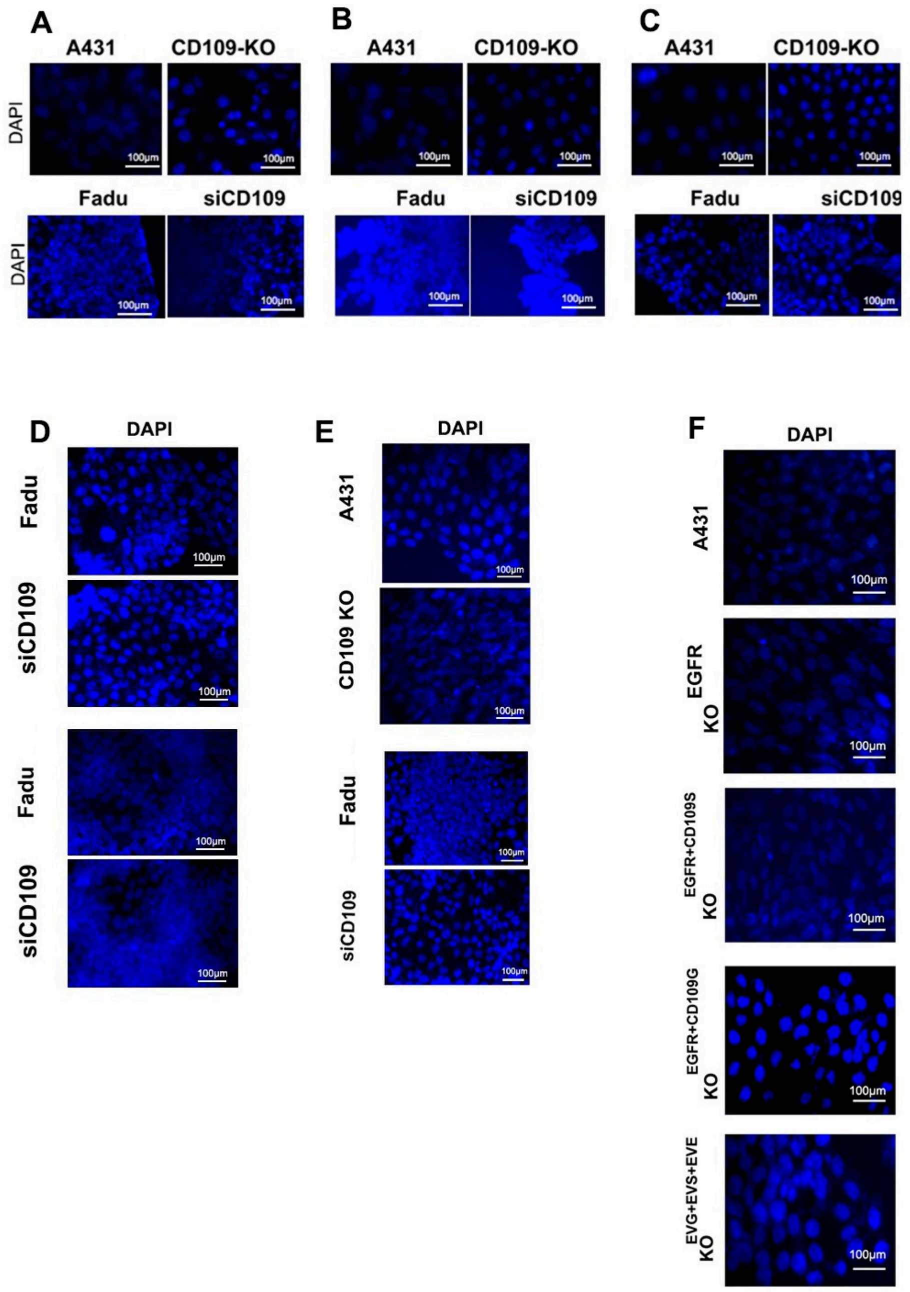

Figure S4: DAPI staining (A) Main figure 3.D. DAPI staining (B) Main figure 3.E DAPI staining (C) Main figure 3.F DAPI staining (D) Main figure 4.D DAPI staining (E) Main figure 4.H DAPI staining (F) Main figure 6.F DAPI staining.

Figure S5: Uncropped WB images.

Figure 3A

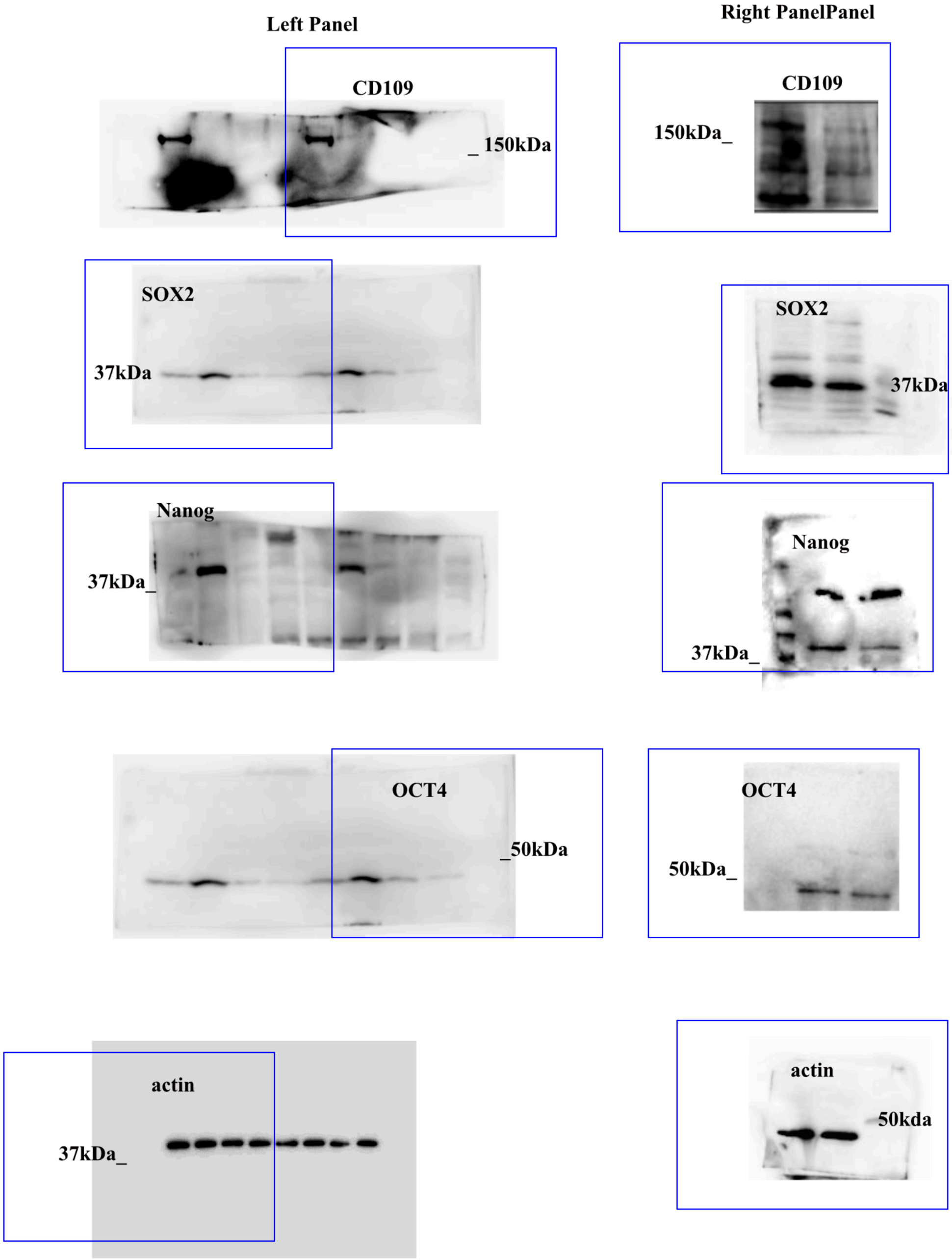

Figure 4C

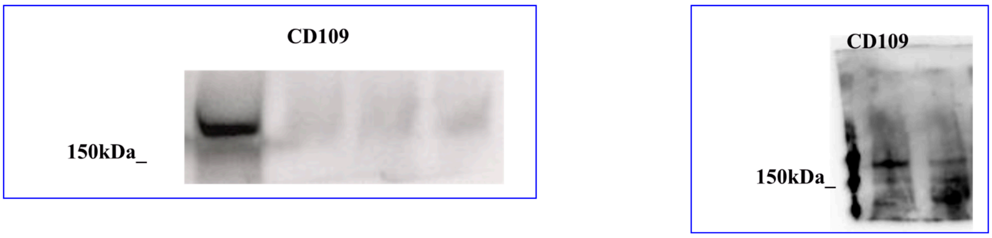

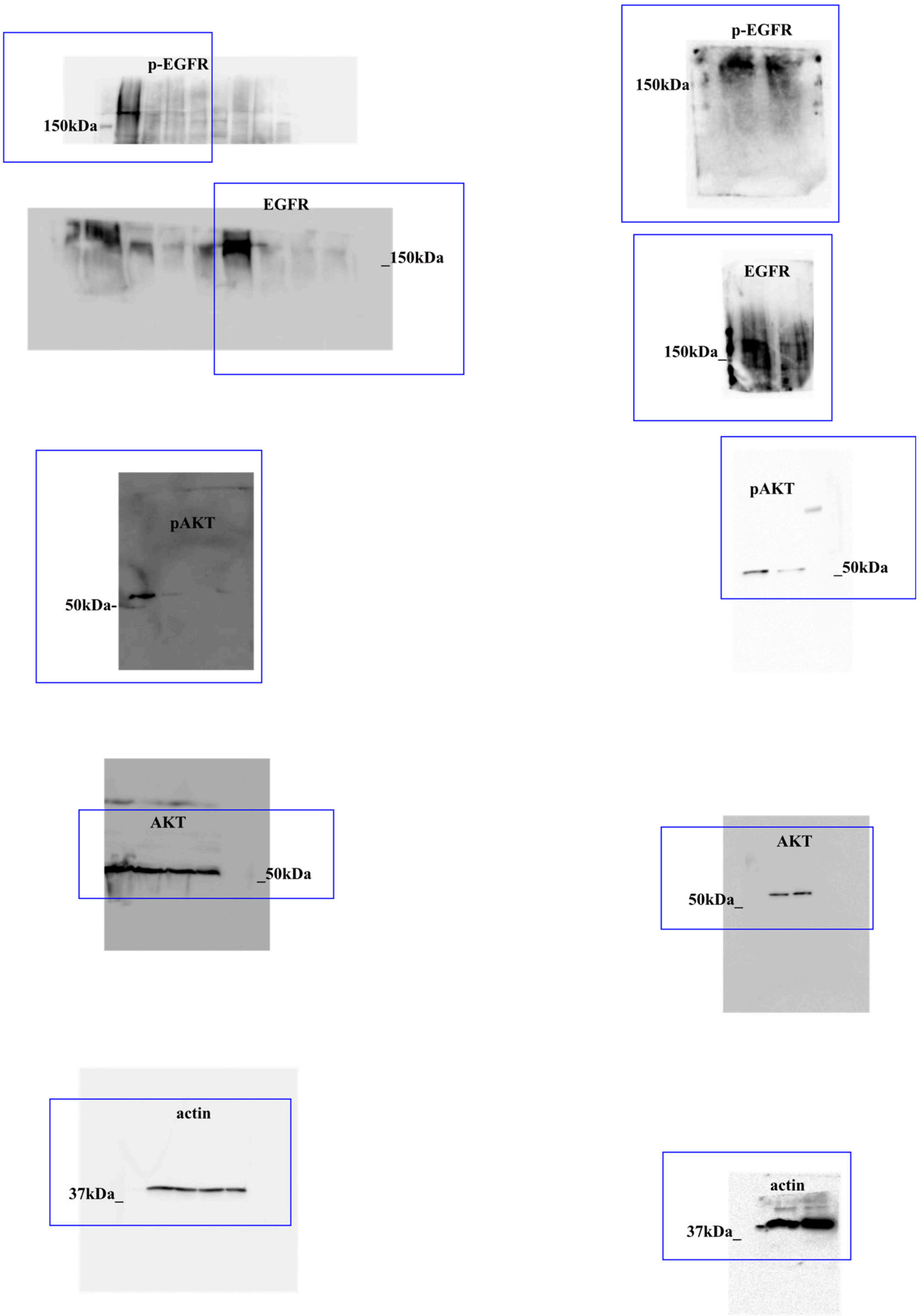
p-EGFR
150kDa
EGFR
_150kDa
pAKT
50kDa-
AKT
_50kDa
actin
37kDa_
p-EGFR
150kDa
EGFR
150kDa_
pAKT
_50kDa
AKT
50kDa_
actin
37kDa_

Figure 4E

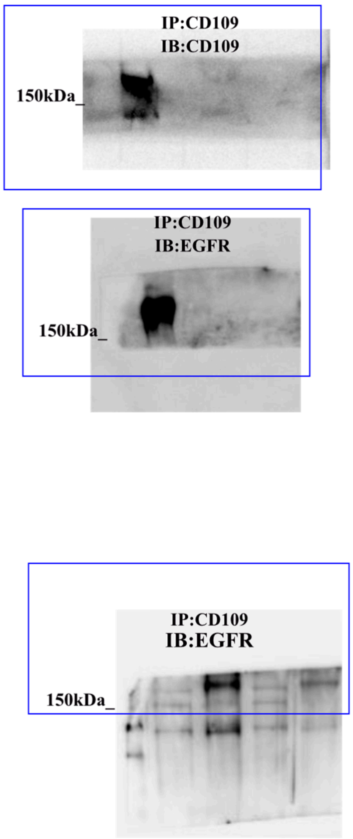

Figure 4F

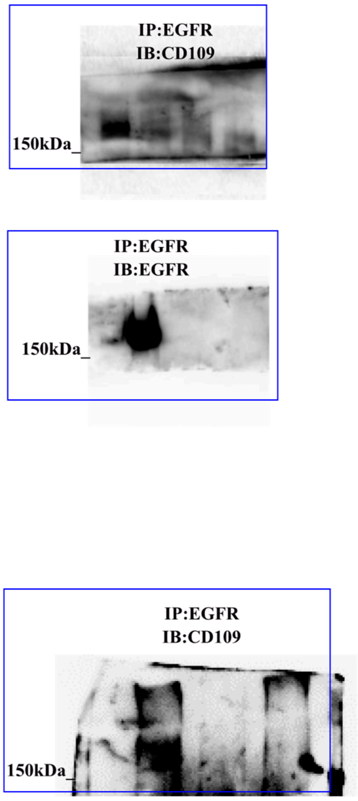

Figure 4G

Input

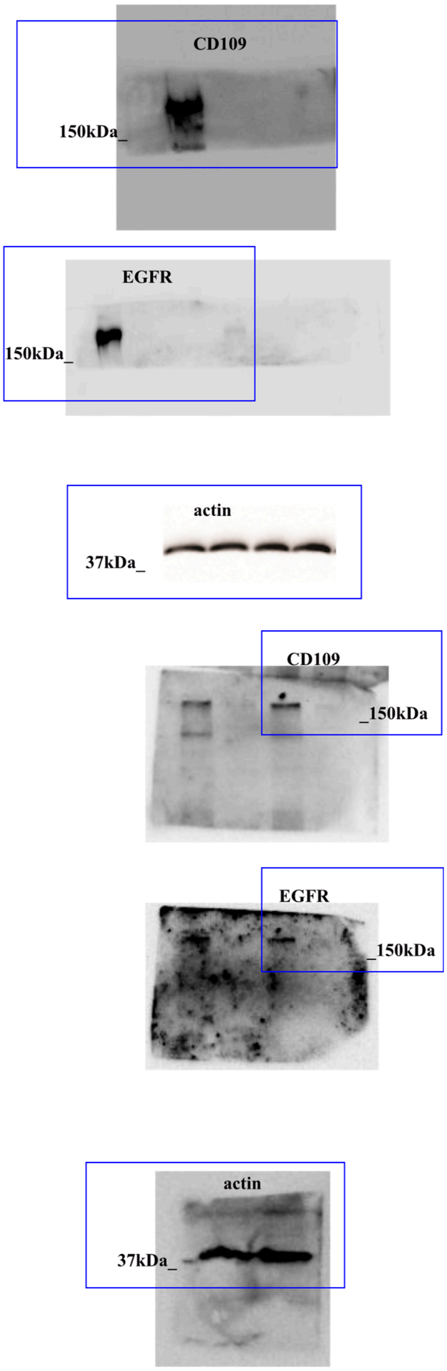

Figure 5A

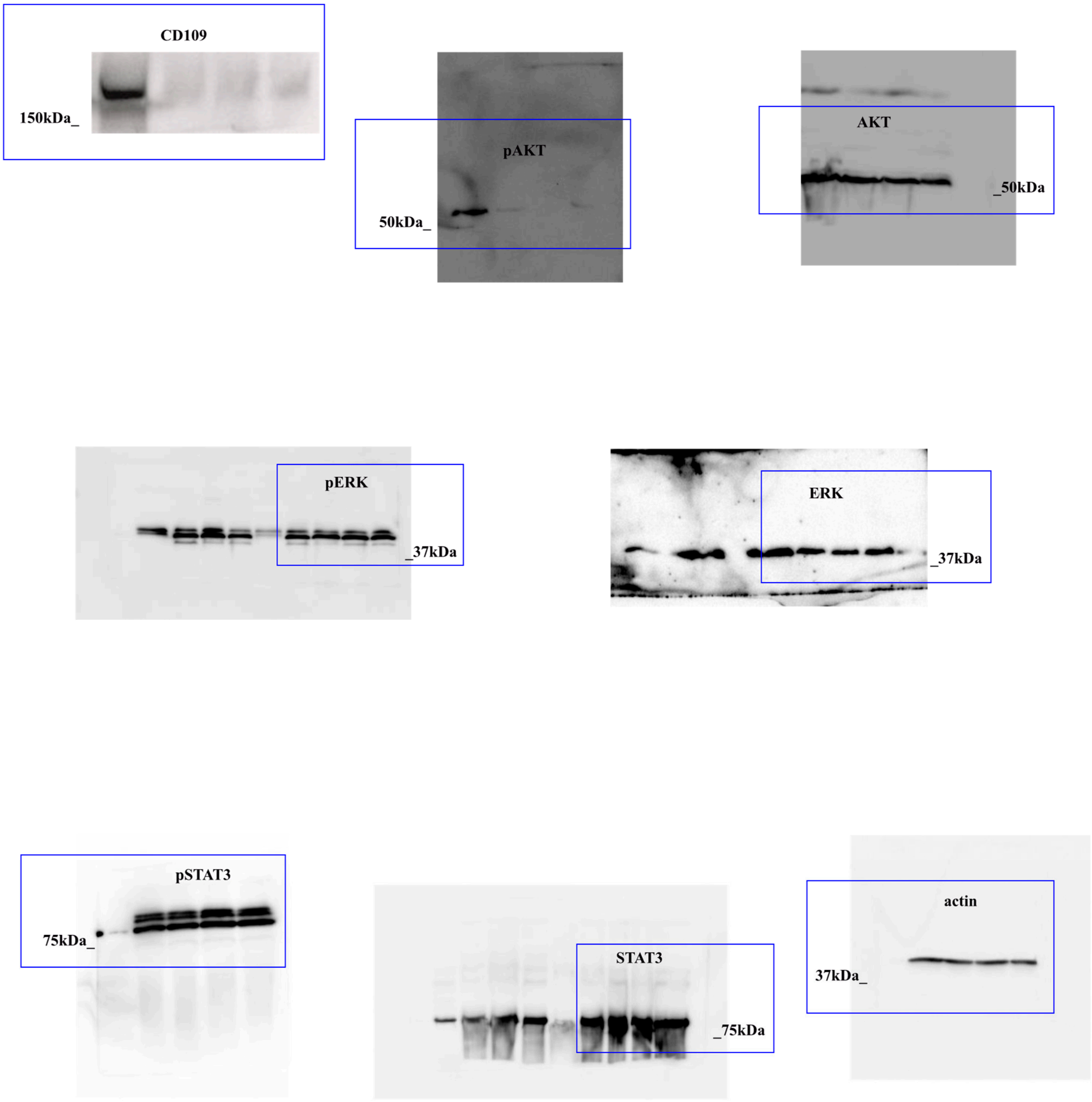
CD109
150kDa_
pAKT
50kDa_
AKT
_50kDa
pERK
_37kDa
ERK
_37kDa
pSTAT3
75kDa_
STAT3
_75kDa
actin
37kDa_

Figure 5C

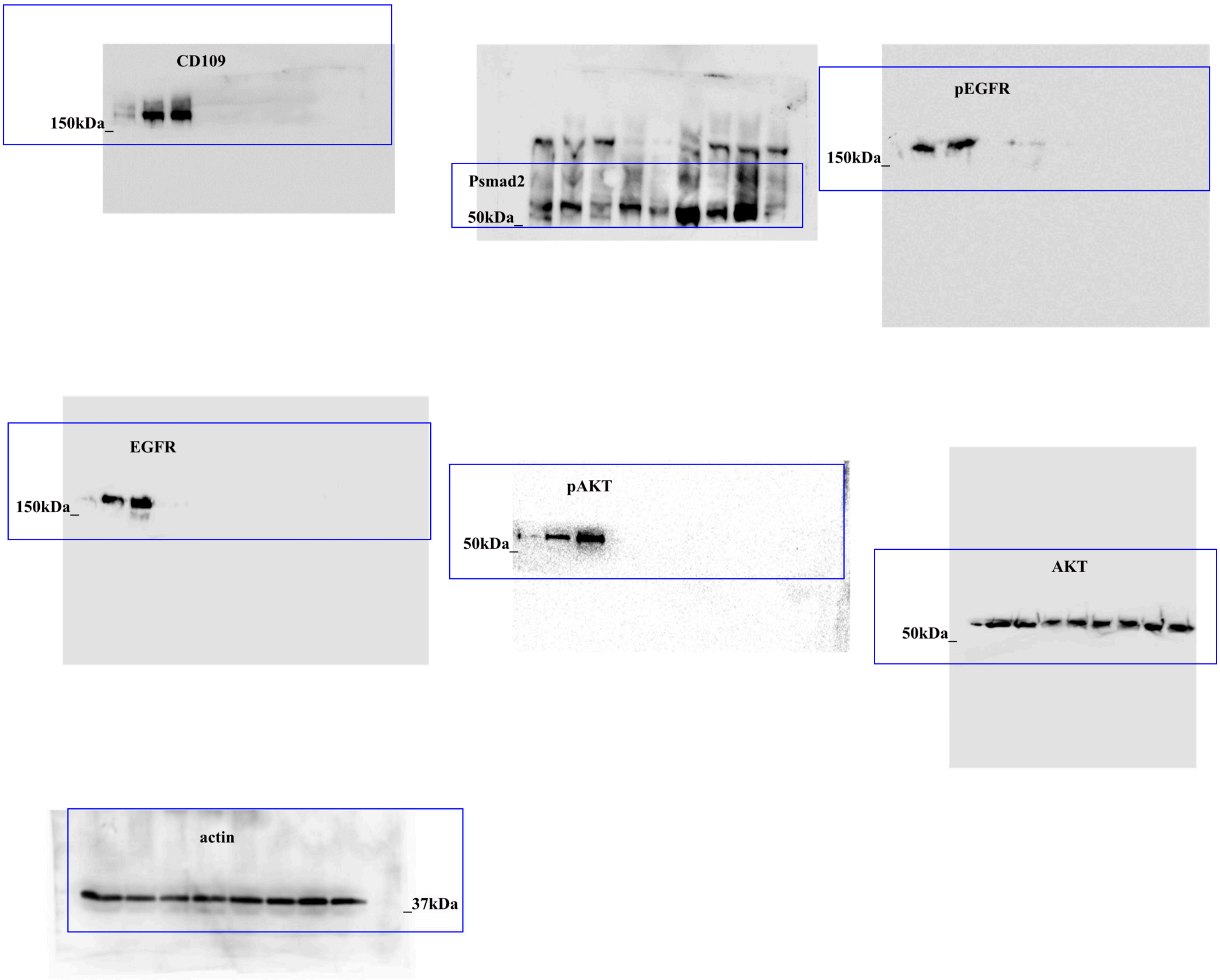
CD109
150kDa_
Psmad2
50kDa_
pEGFR
150kDa_
EGFR
150kDa_
pAKT
50kDa_
AKT
50kDa_
actin
_37kDa

Figure 5F

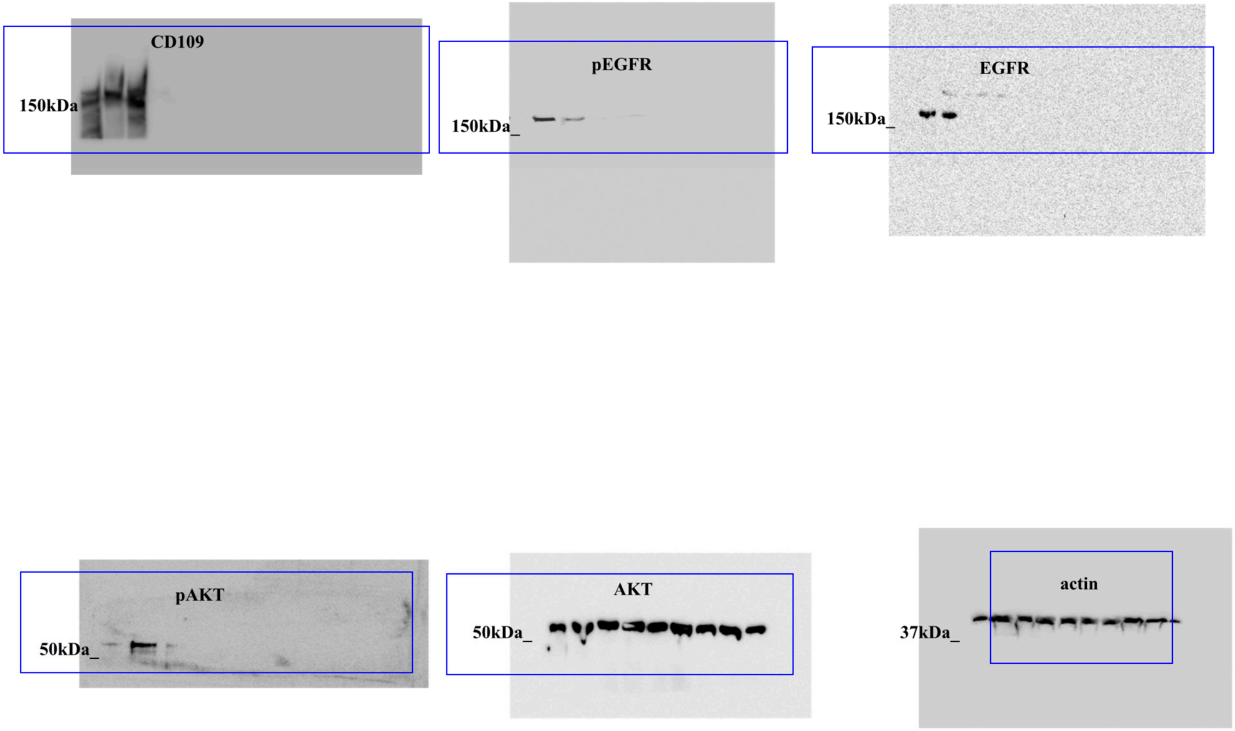

CD109
150kDa
pEGFR
150kDa_
EGFR
150kDa_
pAKT
50kDa_
AKT
50kDa_
actin
37kDa_

Figure 5G

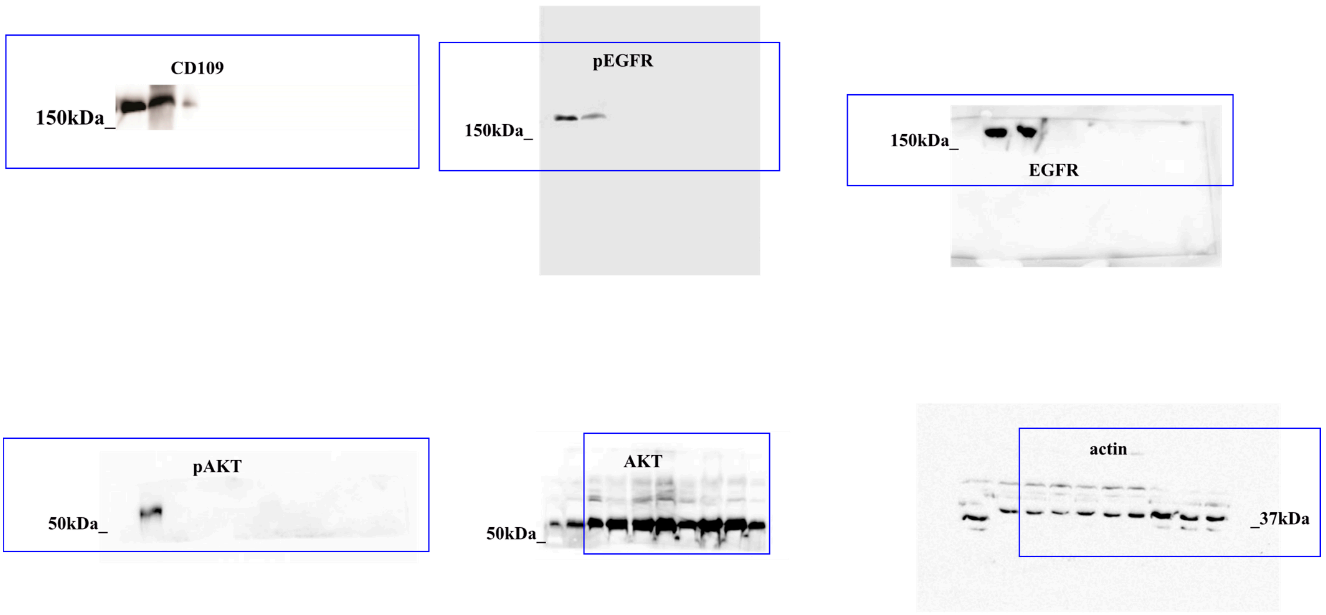

Figure 6B

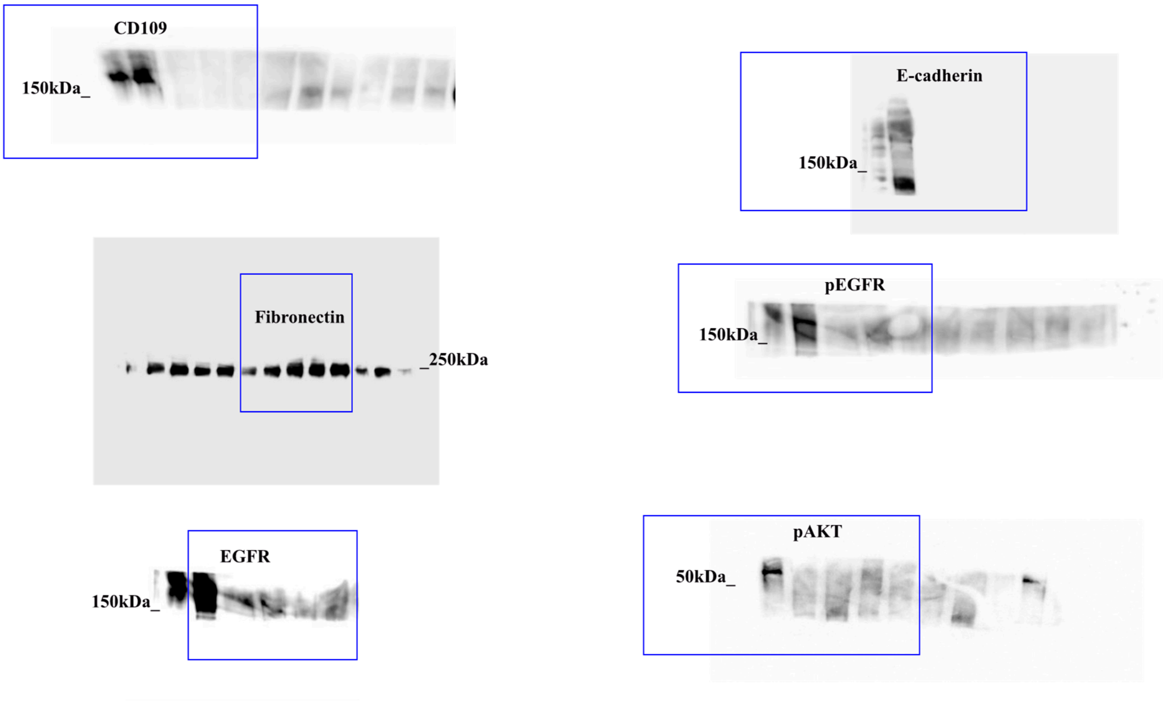

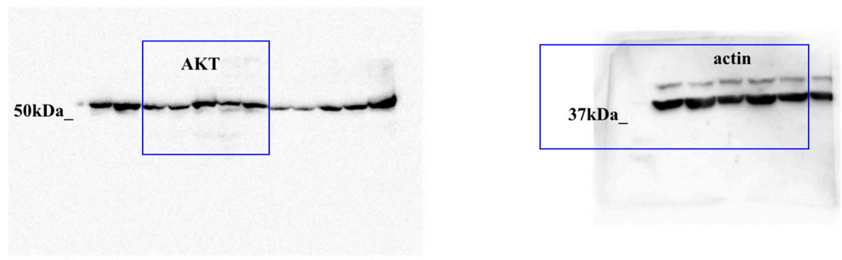
AKT
50kDa_
actin
37kDa_

Figure 6C

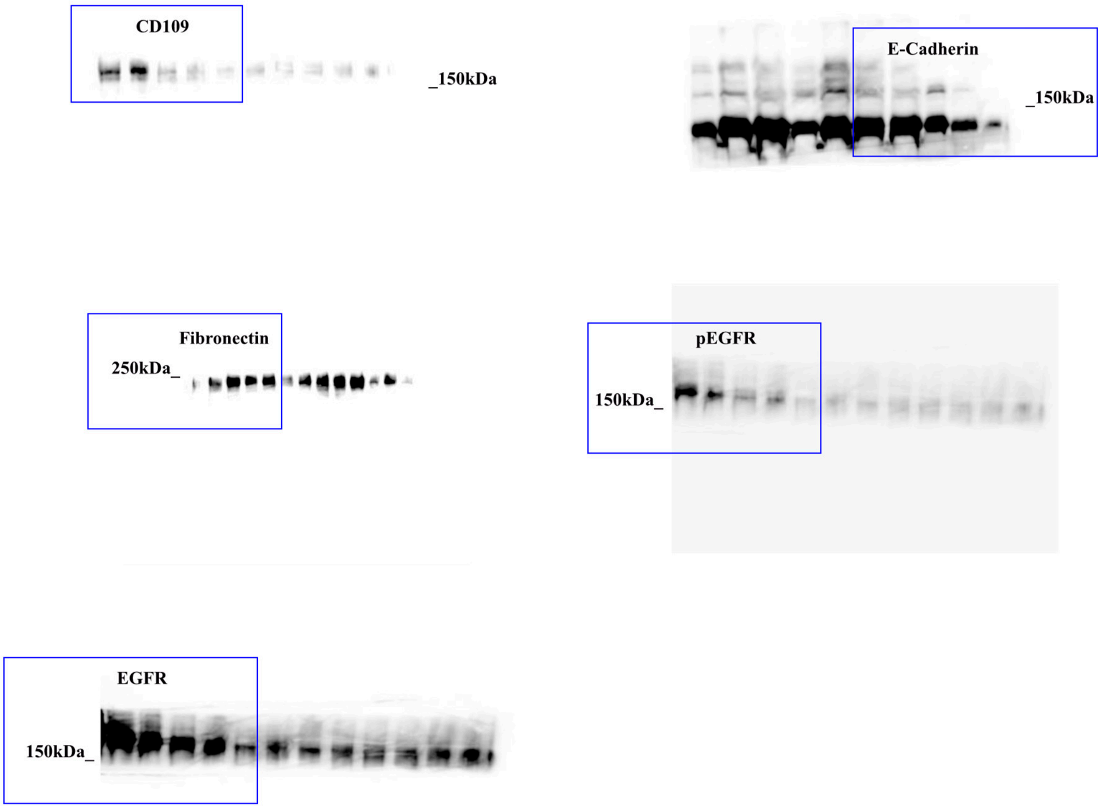
CD109
_150kDa
E-Cadherin
_150kDa
Fibronectin
250kDa_
pEGFR
150kDa_
EGFR
150kDa_

pAKT

50kDa_

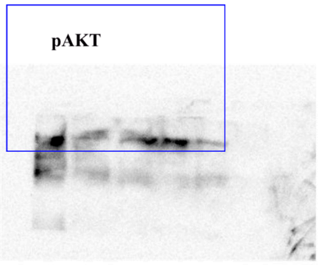

AKT

_50kDa

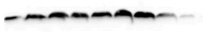

actin

37kDa_

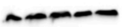

Figure 7A

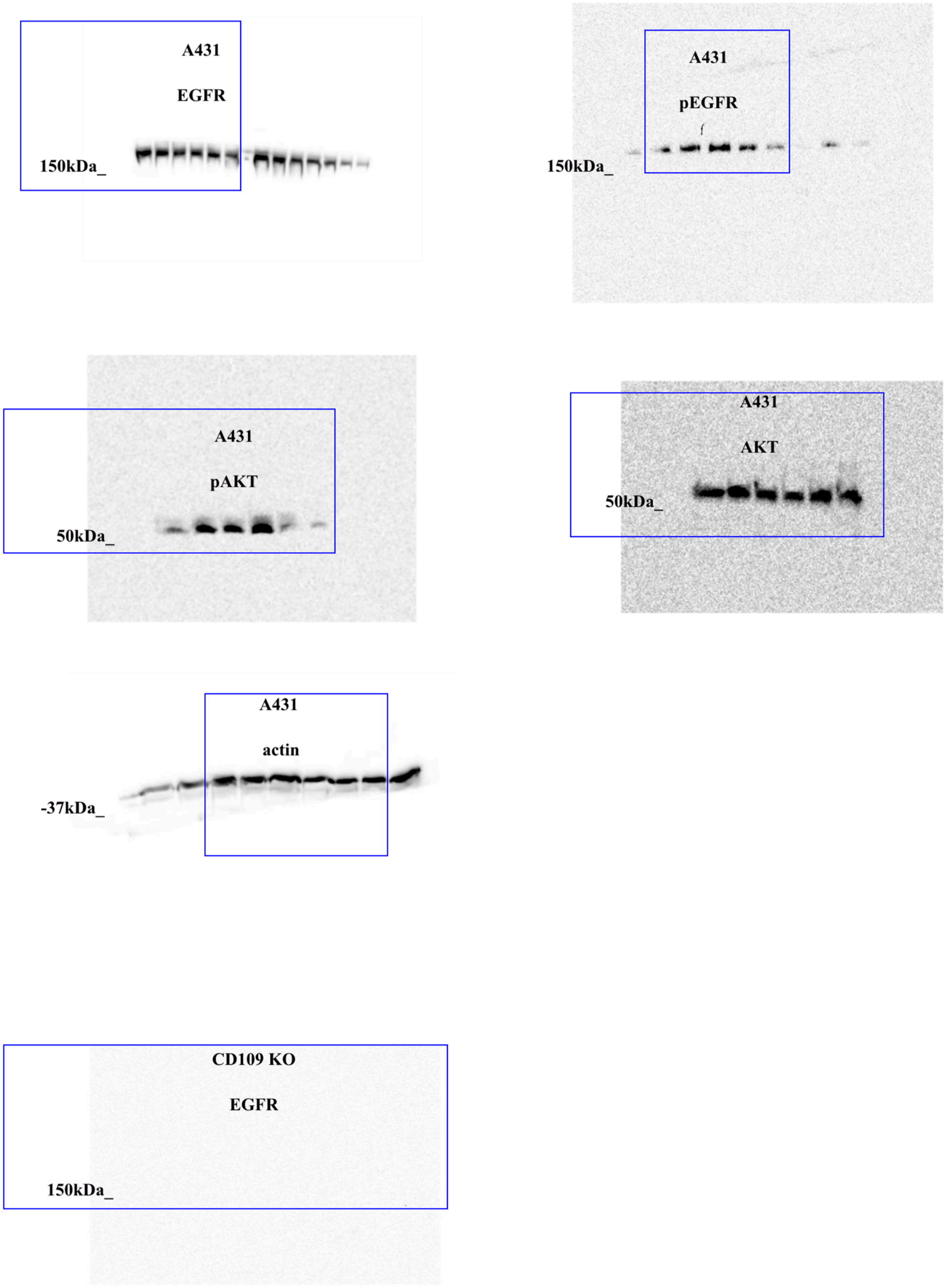
A431
EGFR
150kDa_
A431
pEGFR
150kDa_
A431
pAKT
50kDa_
A431
AKT
50kDa_
A431
actin
-37kDa_
CD109 KO
EGFR
150kDa_

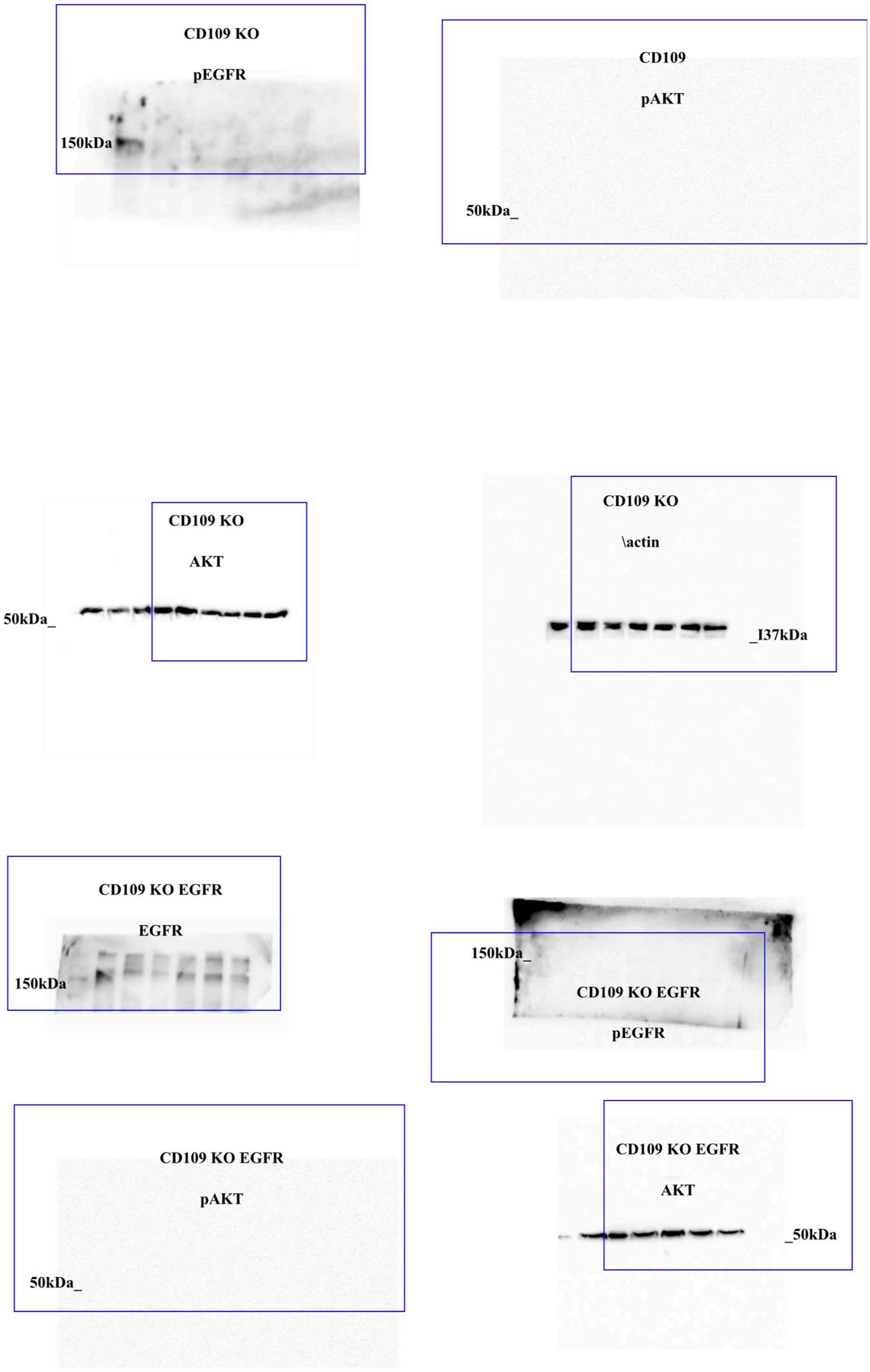
CD109 KO
pEGFR
150kDa
CD109
pAKT
50kDa_
CD109 KO
AKT
50kDa_
CD109 KO
\actin
_I37kDa
CD109 KO EGFR
EGFR
150kDa
150kDa_
CD109 KO EGFR
pEGFR
CD109 KO EGFR
pAKT
50kDa_
CD109 KO EGFR
AKT
_50kDa

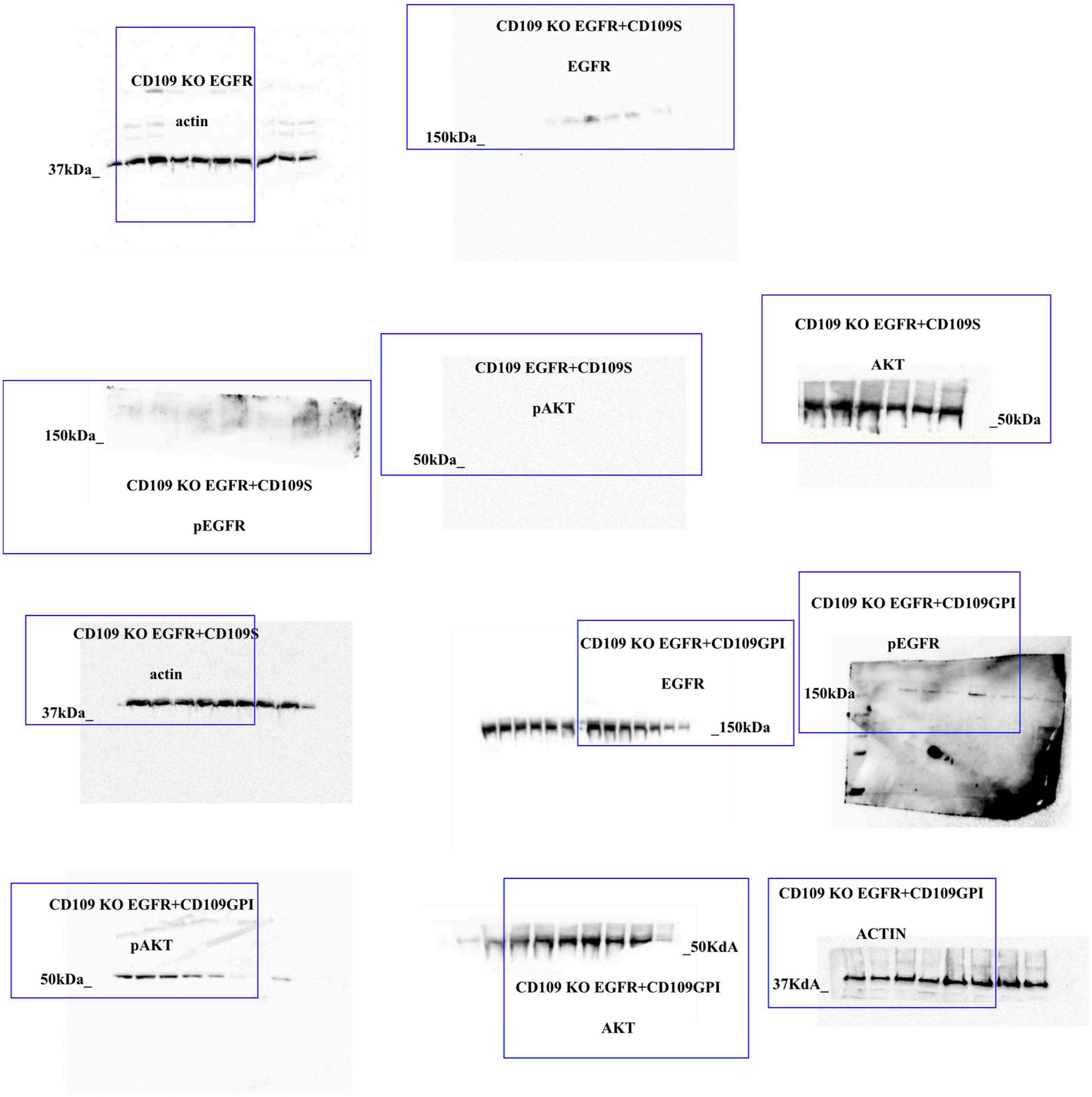
CD109 KO EGFR
actin
37kDa_
CD109 KO EGFR+CD109S
EGFR
150kDa_
CD109 KO EGFR+CD109S
AKT
_50kDa
150kDa_
CD109 KO EGFR+CD109S
pEGFR
CD109 EGFR+CD109S
pAKT
50kDa_
CD109 KO EGFR+CD109S
actin
37kDa_
CD109 KO EGFR+CD109GPI
EGFR
_150kDa
CD109 KO EGFR+CD109GPI
pEGFR
150kDa
CD109 KO EGFR+CD109GPI
pAKT
50kDa_
_50KdA
CD109 KO EGFR+CD109GPI
AKT
CD109 KO EGFR+CD109GPI
ACTIN
37KdA_

Figure S 3D

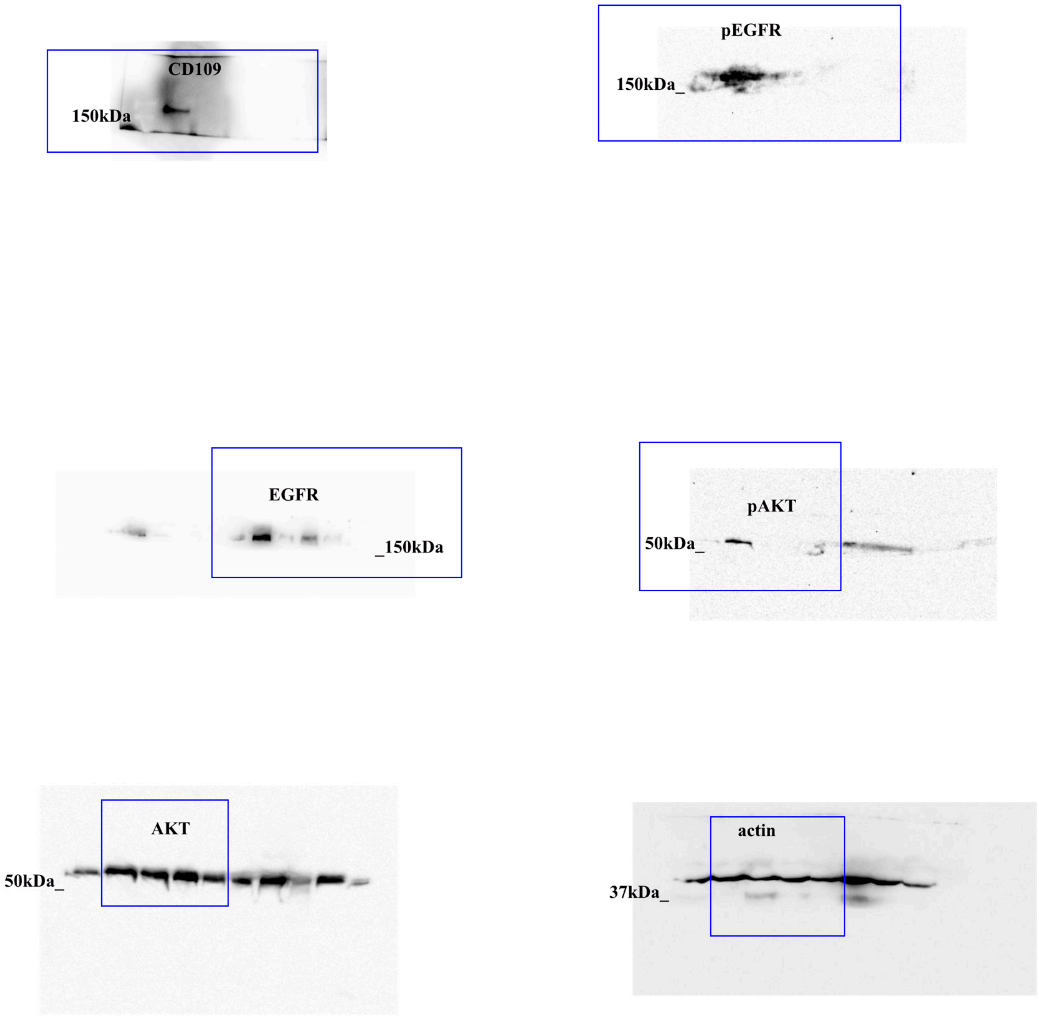
CD109
150kDa
pEGFR
150kDa_
EGFR
_150kDa
pAKT
50kDa_
AKT
50kDa_
actin
37kDa_
